# Supplementary material for: Probing condensate microenvironments with a micropeptide killswitch
Source: Nature. 2025 Jun 4;643(8073):1107–16. doi: 10.1038/s41586-025-09141-5 (PMC12286862; doi:10.1038/s41586-025-09141-5)
Supplement: Supplementary file 1 — Supplementary Figs. 1–15. [file 41586_2025_9141_MOESM1_ESM.pdf]

nature portfolio

https://doi.org/10.1038/s41586-025-09141-5

Supplementary information

Probing condensate microenvironments with a micropeptide killswitch

In the format provided by the authors and unedited

Supplementary Information

Probing condensate microenvironments with a micropeptide killswitch

Yaotian Zhang[1,2], Ida Stöppelkamp[1,2,11], Pablo Fernandez-Pernas[3,11], Melanie Allram[3,11], Matthew Charman[4,5,6,11], Alexandre P. Magalhaes[1], Melanie Piedavent-Salomon[1], Gregor Sommer[1], Yu-Chieh Sung[1], Katrina Meyer[1], Nicholas Grams[5], Edwin Halko[4,6], Shivali Dongre[7], David Meierhofer[1], Michal Malszycki[1], Ibrahim A. Ilik[1], Tugce Aktas[1], Matthew L. Kraushar[1], Nadine Vastenhouw[7], Matthew D. Weitzman[4,5,6,8], Florian Grebien[3,9,10], Henri Niskanen[1$], Denes Hnisz[1$]

[$] Correspondence to: niskanen@molgen.mpg.de, hnisz@molgen.mpg.de

CONTENTS

- Supplementary Figures 1-15

Supplementary Tables 1-3 are uploaded separately

Supplementary Figure 1

a

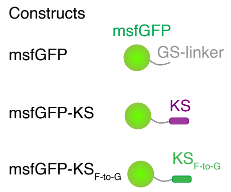

b

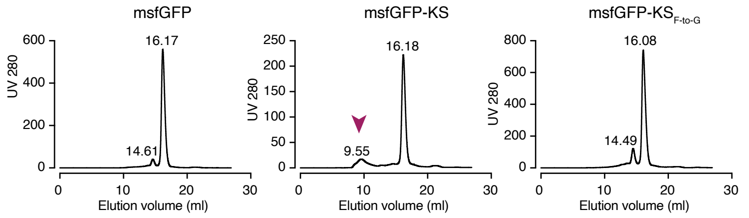

Peak Elution (ml)		Area %	MW (kDa)
P1	14.61	10.5	~54
P2	16.17	88	~30

Peak Elution (ml)		Area %	MW (kDa)
**P1**	**9.55**	**17.5**	**~870**
P2	16.18	78	~30

Peak Elution (ml)		Area %	MW (kDa)
P1	14.49	20	~54
P2	16.18	78	~30

SEC chromatograms of the recombinant msfGFP proteins eluted on Superdex 200 column with SEC buffer containing 50 mM Tris pH 7.5, 500 mM NaCl.

c

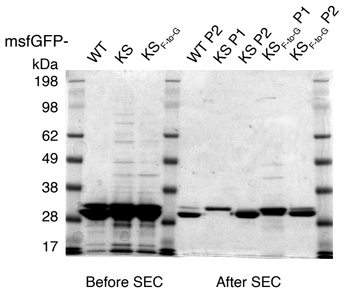

Coomassie gel of purified proteins before and after SEC elution.

d

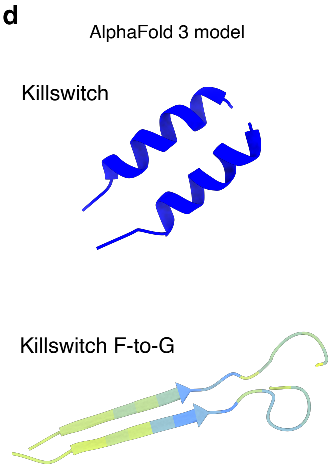

e

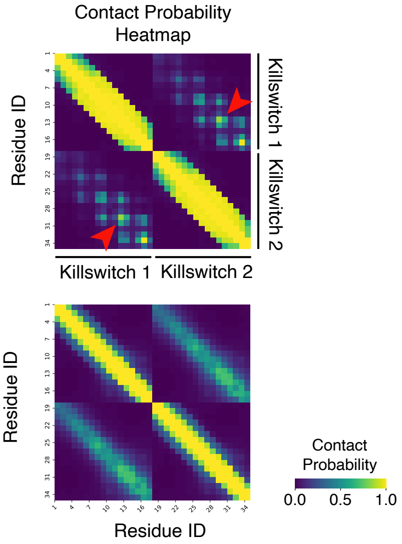

Supplementary Figure 1. Self-association of the killswitch is dependent on phenylalanines.

a. Schematic models of msfGFP constructs fused with C-terminal KS and $KS_{F\text{-to-}G}$.

b. Size-exclusion chromatography (SEC) chromatograms of recombinant msfGFP, msfGFP-KS and msfGFP-$KS_{F\text{-to-}G}$. Elution volumes and estimated sizes of proteins eluted in peaks 1 and 2 (P1, P2) are shown below.

c. Coomassie gel of purified proteins before and after size-exclusion chromatography (SEC). P1: peak 1, P2: peak 2 shown in **b**. This experiment was performed once.

d. (top) AlphaFold 3 model of a killswitch dimer; and (bottom) Alphafold 3 model of a killswitch F-to-G mutant dimer. The depth of the color is proportional to the model confidence. Dark blue is high confidence prediction, dim yellow is low confidence.

e. Contact probability heatmap of the killswitch and killswitch F-to-G mutant dimer. In this analysis, the two sequences that make up the dimer are displayed next to each other as a tandem sequence (i.e. Killswitch 1 and Killswitch 2; in total consisting of 34 amino acids), on each axis. The contact probability of each residue with any other residues within the same killswitch or the other half of the dimer are shown as a heatmap. Red arrows highlight the position of the 3 phenylalanines (positions: 12-14, and 29-31), where the contact probability is the highest between the killswitch dimers. The contacts are lost in the F-to-G mutant, shown in the bottom.

Supplementary Figure 2

a

	Description	Scientific Name	Max Score	Total Score	Query Cover	E value	Per. Ident	Acc. Len	Accession
☑	olfactory receptor [Homo sapiens]	Homo sapiens	27.8	27.8	94%	1.7	40.74%	216	AAK95009.1
☑	OR5D16 [Homo sapiens]	Homo sapiens	27.8	27.8	94%	1.7	40.74%	282	ALI87696.1
☑	olfactory receptor 5D16 [Homo sapiens]	Homo sapiens	27.8	27.8	94%	1.7	40.74%	328	NP_001005496.1
☑	hCG401289, isoform CRA_e [Homo sapiens]	Homo sapiens	26.1	26.1	47%	6.8	75.00%	148	EAX09479.1
☑	pyridoxal kinase [Homo sapiens]	Homo sapiens	26.1	26.1	47%	6.8	75.00%	148	KAI4004233.1
☑	pyridoxal kinase isoform X2 [Homo sapiens]	Homo sapiens	26.1	26.1	47%	6.9	75.00%	373	XP_047296957.1
☑	NADH dehydrogenase subunit 2 [Homo sapiens]	Homo sapiens	25.7	25.7	82%	9.8	52.94%	347	ADI92818.1
☑	chromosome condensation protein G [Homo sapiens]	Homo sapiens	25.2	25.2	47%	14	87.50%	1015	AAG30732.1
☑	alternative protein RBM28 [Homo sapiens]	Homo sapiens	24.8	24.8	41%	18	71.43%	47	CCQ43582.1
☑	voltage-gated calcium channel alpha 1 subunit [Homo sapiens]	Homo sapiens	24.8	90.4	41%	19	71.43%	125	AAD08651.1

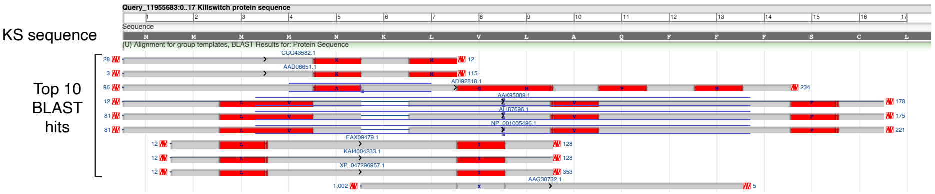

Alignment summary of the top 10 BLAST hits ranked by E-values on BLAST protein search for the Killswitch protein sequence. The gray-shaded residues are the matching residues to the KS sequence. The red-shaded residues are mismatch.

b

Killswitch ("KS"): MMMMNKLVLAQFFFSCL

TDP43 hydrophobic patch ("TDP43 HP"): INPAMMAAAQAALQSSWGMMGMLASQ

c

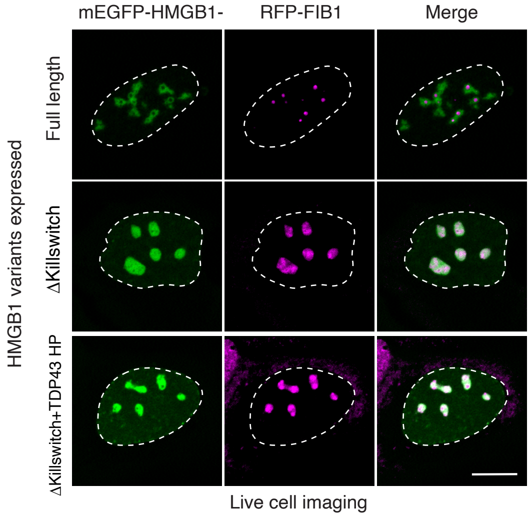

d

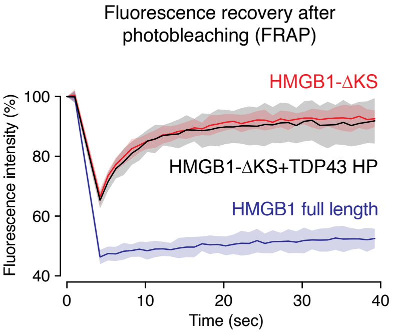

e

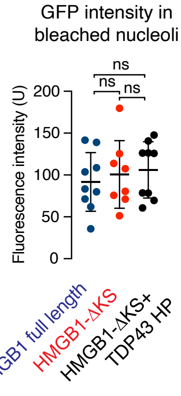

f

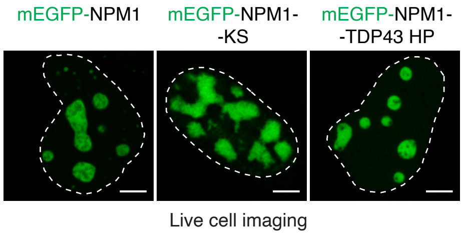

g

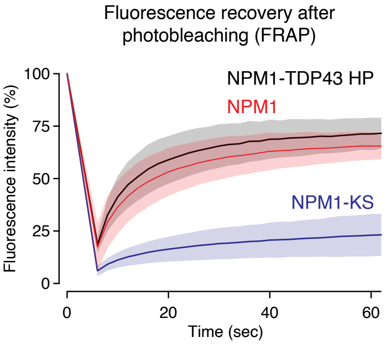

h

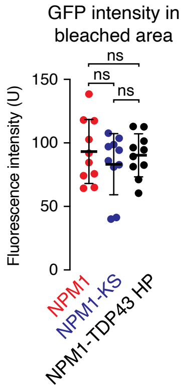

Supplementary Figure 2. The killswitch has no "homolog" in the human proteome, and it affects nucleoli differently than the TDP43 hydrophobic patch.

a. Alignment summary of the top 10 BLAST hits ranked by E-values on BLAST protein search for the KS protein sequence. The gray-shaded residues are the matching residues to the KS sequence. The red-shaded are mismatch.

b. Protein sequences of the KS and TDP43 hydrophobic patch (TDP43 HP).

c. Live cell fluorescence microscopy images of U2OS cells expressing ectopic mEGFP-fsHMGB1, HMGB1 without the KS (fsHMGB1ΔKS) or fsHMGB1ΔKS with TDP43 hydrophobic patch (TDP43 HP) and RFP-FIB1. The cell nucleus is highlighted with a dashed white line contour. This experiment was performed once. Scale bar: 10µm.

d. Fluorescence recovery after photobleaching (FRAP) of mEGFP-fsHMGB1. Line displays the mean and shaded color +/- standard deviation. fsHMGB1, n = 9; -ΔKS, n = 8; -ΔKS+TDP43 HP, n = 9.

e. Mean GFP fluorescence of bleached area. Data are presented as mean values +/- SD. *P*-values are from Tukey's multiple comparison test after one-way ANOVA, n of samples as in panel d. $P_{(\text{HMGB1-fulllength vs HMGB1-}\Delta\text{KS})} = 0.87$, $P_{(\text{HMGB1-fulllength vs HMGB1-}\Delta\text{KS+TDP43HP})} = 0.68$, $P_{(\text{HMGB1-}\Delta\text{KS vs HMGB1-}\Delta\text{KS+TDP43HP})} = 0.95$. fsHMGB1.

f. Live cell fluorescence microscopy images of U2OS cells expressing ectopic EGFP-NPM1, -NPM1 with C-terminal KS or TDP43 hydrophobic patch. The cell nucleus is highlighted with a dashed white line contour. Scale bar: 5µm.

g. FRAP of EGFP-NPM1. Line displays the mean and shaded color +/- standard deviation. n = 10 for all samples.

h. Mean GFP fluorescence of bleached area. Data are presented as mean values +/- SD. *P*-values from Tukey's post-hoc test after one-way ANOVA. $P_{(\text{NPM1 vs NPM1-KS})} = 0.58$, $P_{(\text{NPM1 vs NPM1-TDP43-HP})} = 0.95$, $P_{(\text{NPM1-KS vs NPM1-TDP43-HP})} = 0.76$. n = 10 cells for all samples from two biologically independent experiments.

Supplementary Figure 3

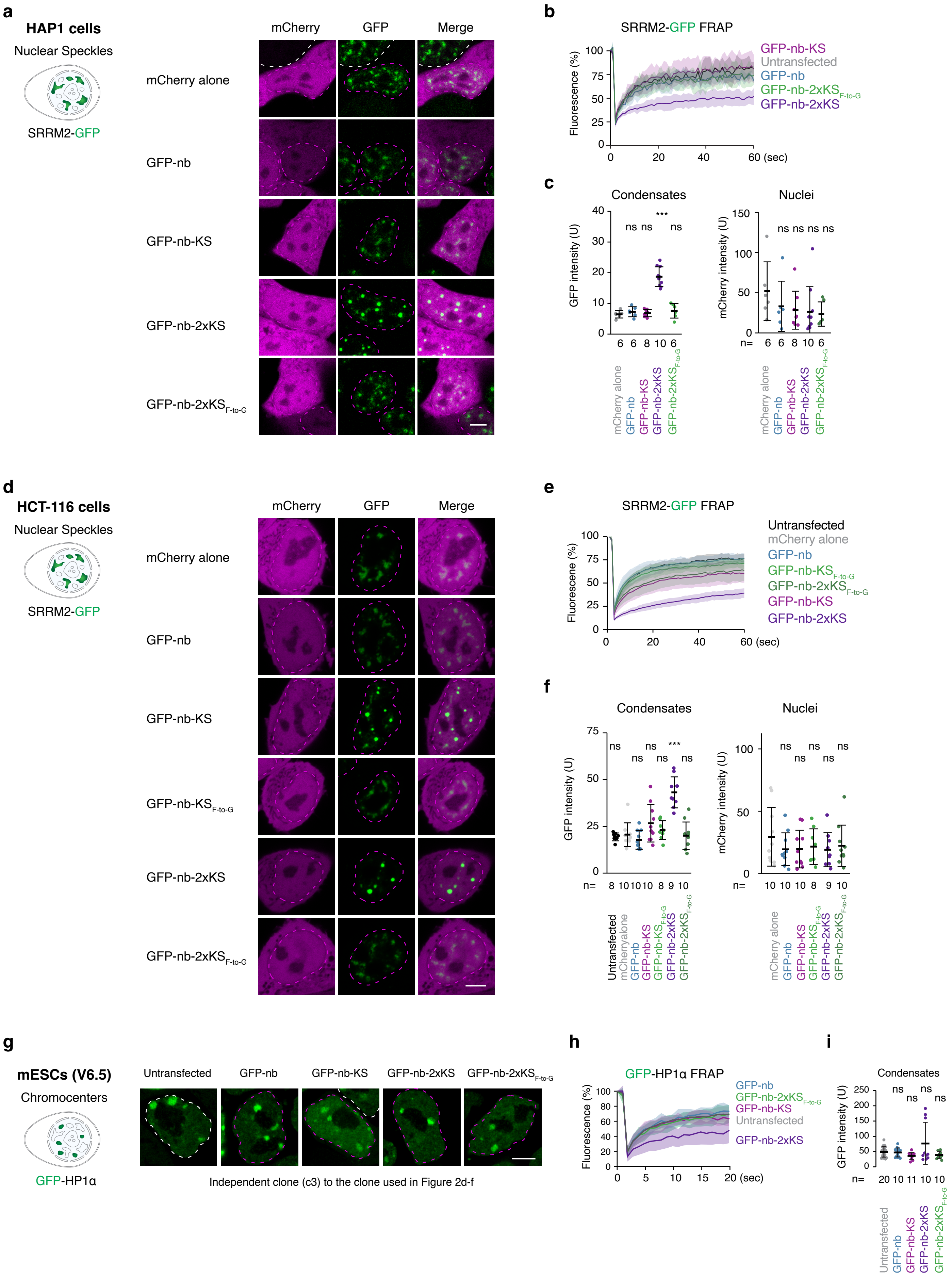

Supplementary Figure 3. Nanobody-based killswitch recruitment system arrests dynamics of endogenous nuclear speckle protein SRRM2 and chromocenter associated HP1α.

a. Live cell fluorescence microscopy images of HAP1 cells expressing SRRM2-GFP from endogenous locus and ectopic GFP-nb constructs with cleaved mCherry reporter. The cell nucleus is highlighted with a dashed line contour where magenta color represents transfected cell expressing GFP-nb. Scale bar: 5μm.

b. FRAP of SRRM2-GFP in HAP1 cells. Line displays the mean and shaded color +/- standard deviation, n of samples is same as in **c**.

c. Quantification of (left) mean GFP fluorescence of bleached condensates and (right) mean mCherry fluorescence of nuclei of examined HAP1 cells. Mean is shown as a black line and whiskers represent standard deviation. *P*-values are from Tukey's post-hoc test versus mCherry-alone after one-way ANOVA. For GFP intensity plot, $P_{(\text{GFP-nb})} = 0.97$, $P_{(\text{GFP-nb-KS})} = 0.997$, $P_{(\text{GFP-nb-2xKS})} = <0.0001$, $P_{(\text{GFP-nb-2xKS-F-to-G})} = 0.90$. For mCherry intensity plot, $P_{(\text{GFP-nb})} = 0.78$, $P_{(\text{GFP-nb-KS})} = 0.55$, $P_{(\text{GFP-nb-2xKS})} = 0.42$, $P_{(\text{GFP-nb-2xKS-F-to-G})} = 0.43$.

d. Live cell fluorescence microscopy images of HCT-116 cells expressing SRRM2-GFP from endogenous locus and ectopic GFP-nb constructs with cleaved mCherry reporter. The cell nucleus is highlighted with a dashed line contour where magenta color represents transfected cell expressing GFP-nb. Scale bar: 5μm.

e. FRAP of SRRM2-GFP in HCT-116 cells. Line displays the mean and shaded color +/- standard deviation. n of samples is same as in **f.**

f. Quantification of (left) mean GFP fluorescence of bleached condensates and (right) mean mCherry fluorescence of nuclei of examined HCT-116 cells. Mean is shown as a black line and whiskers represent standard deviation. *P*-values are from Tukey's post-hoc test versus mCherry-alone after one-way ANOVA. For GFP intensity plot, $P_{(\text{untransfected})} = 0.9998$, $P_{(\text{GFP-nb})} = 0.97$, $P_{(\text{GFP-nb-KS})} = 0.43$, $P_{(\text{GFP-nb-KS-F-to-G})} = 0.99$, $P_{(\text{GFP-nb-2xKS})} = <0.001$, $P_{(\text{GFP-nb-2xKS-F-to-G})} = 1.0$. For mCherry intensity plot, $P_{(\text{GFP-nb})} = 0.76$, $P_{(\text{GFP-nb-KS})} = 0.77$, $P_{(\text{GFP-nb-KS-F-to-G})} = 0.92$, $P_{(\text{GFP-nb-2xKS})} = 0.75$, $P_{(\text{GFP-nb-2xKS-F-to-G})} = 0.93$.

g. Live cell fluorescence microscopy images of mouse ES cells expressing GFP-HP1α from endogenous locus and ectopic GFP-nb constructs with cleaved mCherry reporter. Scale bar: 5 μm.

h. FRAP of GFP-HP1α in mouse ES cells. Line displays the mean and shaded color +/- standard deviation. n of samples is the same as in **i.**

i. Quantification of mean GFP fluorescence of bleached chromocenters in mES cells. *P*-values are from Dunnett's multiple comparison test versus untransfected control after one-way ANOVA. $P_{(\text{GFP-nb})} = 0.995$, $P_{(\text{GFP-nb-KS})} = 0.59$, $P_{(\text{GFP-nb-2xKS})} = 0.11$, $P_{(\text{GFP-nb-2xKS-F-to-G})} = 0.70$. Mean is shown as a black line and whiskers represent standard deviation.

Supplementary Figure 4

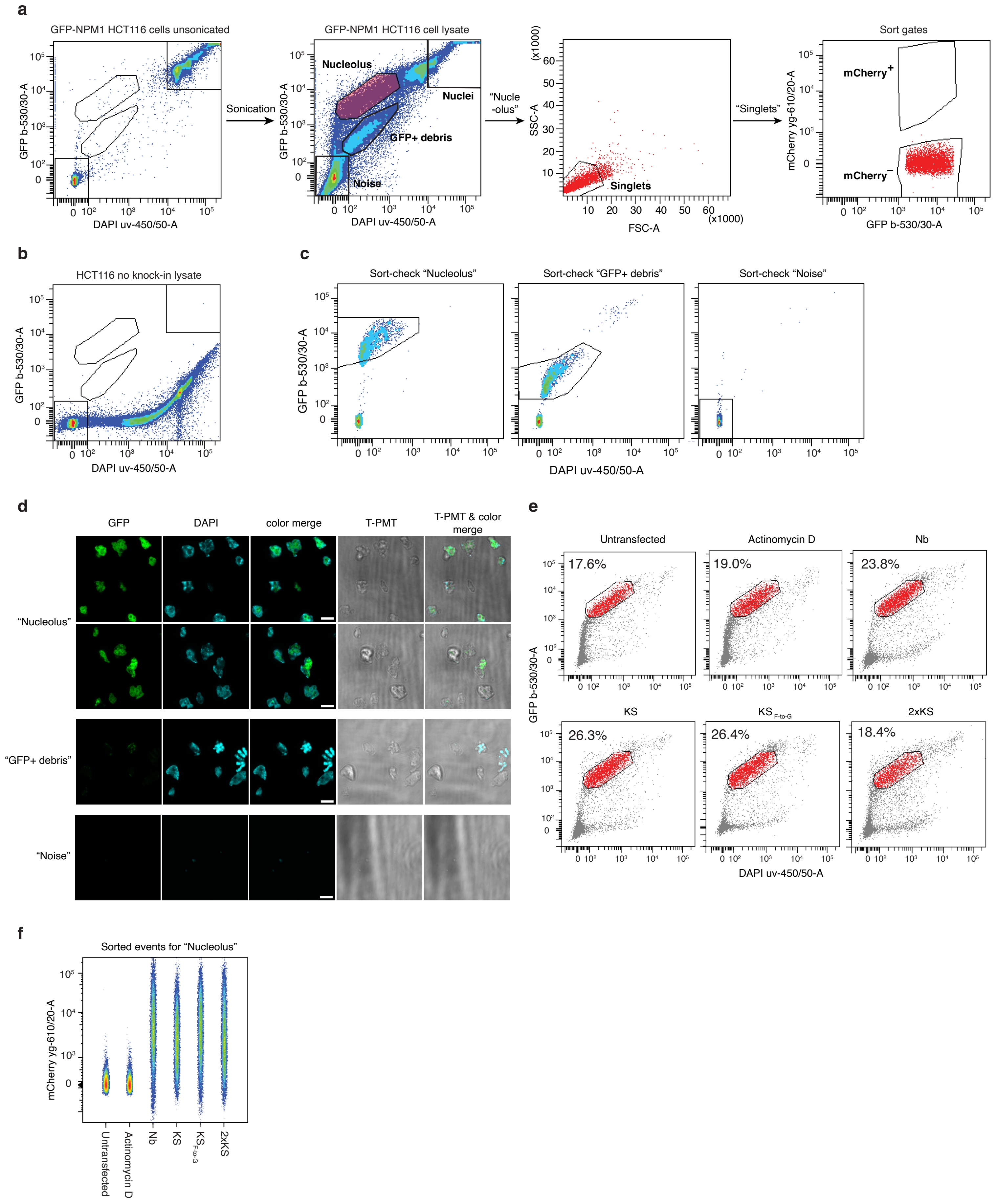

Supplementary Figure 4. FACS strategy and control plots of NuFANCI for nucleoli

a. Representative FACS plots showing the general sorting strategy for isolating nucleoli from HCT116 GFP-NPM1 cells.

b. FACS plot of lysates from HCT116 cells without GFP-NPM1 knock-in, analyzed using the same settings as in **a**.

c. Post-sort analysis of three populations: “Nucleolus”, “GFP+ debris”, and “Noise” from the HCT116 GFP-NPM1 lysate.

d. Representative images of particles collected from the three populations checked in **c**.

e. FACS plots of HCT116 GFP-NPM1 lysates with conditions of untransfected, ActD-treated, transfected with GFP-nb, GFP-nb-KS, GFP-nb- $KS_{F\text{-}to\text{-}G}$, and GFP-nb-2xKS. The percentage of “Nucleolus” events among total events is indicated in the top-left corner of each plot.

f. Fluorescence intensity of mCherry expression in the “Nucleolus” population for each condition described in **e**.

Supplementary Figure 5

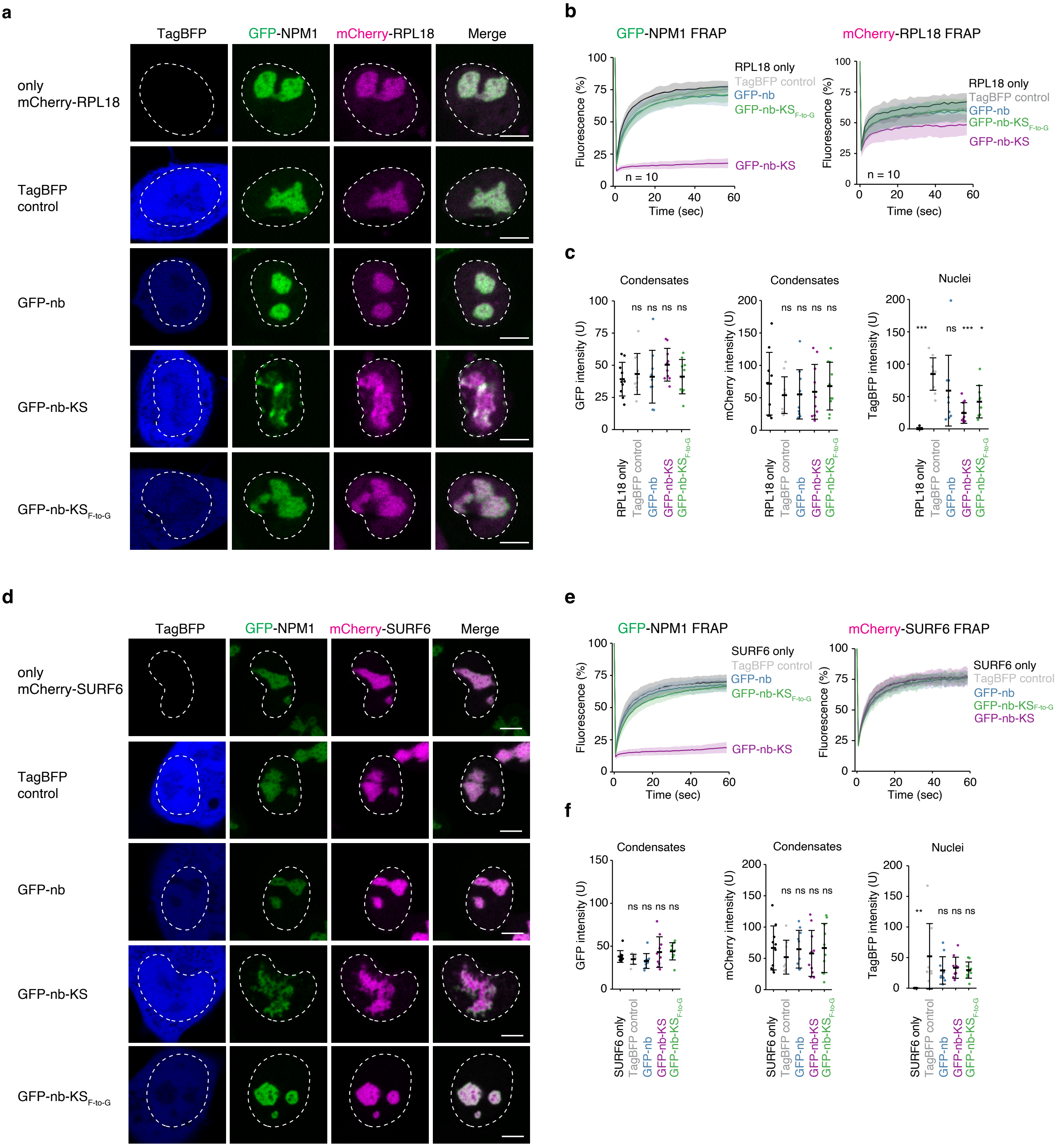

Supplementary Figure 5. Immobilizing GFP-NPM1 inhibits the dynamics of RPL18 but not SURF6 in the nucleolus.

a. Live cell fluorescence microscopy images of HCT-116 cells expressing GFP-NPM1 from endogenous locus and ectopic GFP-nb constructs with cleaved TagBFP reporter and mCherry-RPL18. Scale bar = 5 μm

b. FRAP of GFP-NPM1 and mCherry-SURF6 in cells expressing indicated GFP-nb constructs. Line displays the mean and shaded color +/- SD.

c. Quantification of mean GFP (left) and mCherry (middle) fluorescence of bleached condensates and mean TagBFP (right) fluorescence of nuclei of examined HCT-116 cells. Mean is shown as a black line and whiskers represent standard deviation. *P*-values are from Tukey's post-hoc test versus mCherry-RPL18-only for GFP and mCherry intensity plot and versus TagBFP-control for TagBFP intensity plot after one-way ANOVA. For GFP intensity plot, $P(_{\text{TagBFP control}}) = 0.98$, $P(_{\text{GFP-nb}}) = 0.999$, $P(_{\text{GFP-nb-KS}}) = 0.49$, $P(_{\text{GFP-nb-KS-F-to-G}}) = 0.999$; for mCherry intensity plot, $P(_{\text{TagBFP control}}) = 0.85$, $P(_{\text{GFP-nb}}) = 0.88$, $P(_{\text{GFP-nb-KS}}) = 0.95$, $P(_{\text{GFP-nb-KS-F-to-G}}) = 1.0$; for TagBFP intensity plot, $P(_{\text{RPL18 only}}) = <0.001$, $P(_{\text{GFP-nb}}) = 0.33$, $P(_{\text{GFP-nb-KS}}) = <0.001$, $P(_{\text{GFP-nb-KS-F-to-G}}) = 0.02$. "n" represents number of cells as in **b** examined over one experiment. The experiment was repeated independently three times with similar results.

d. Live cell fluorescence microscopy images of HCT-116 cells expressing GFP-NPM1 from endogenous locus and ectopic GFP-nb constructs with cleaved TagBFP reporter and mCherry-SURF6. Scale bar = 5 μm

e. FRAP of GFP-NPM1 and mCherry-SURF6 in cells expressing indicated GFP-nb constructs. Line displays the mean and shaded color +/- SD. n = 10 for all cases except 8 for TagBFP control.

f. Quantification of mean GFP and mCherry fluorescence of bleached condensates and mean TagBFP fluorescence of nuclei of examined HCT-116 cells. Mean is shown as a black line and whiskers represent standard deviation. *P*-values are from Tukey's post-hoc test versus mCherry-SURF6-only for GFP and mCherry intensity plot and versus TagBFP-control for TagBFP intensity plot after one-way ANOVA. For GFP intensity plot, $P(_{\text{TagBFP control}}) = 0.97$, $P(_{\text{GFP-nb}}) = 0.82$, $P(_{\text{GFP-nb-KS}}) = 0.83$, $P(_{\text{GFP-nb-KS-F-to-G}}) = 0.73$; for mCherry intensity plot, $P(_{\text{TagBFP control}}) = 0.89$, $P(_{\text{GFP-nb}}) = 1.0$, $P(_{\text{GFP-nb-KS}}) = 0.98$, $P(_{\text{GFP-nb-KS-F-to-G}}) = 1.0$; for TagBFP intensity plot, $P(_{\text{SURF6 only}}) = 0.001$, $P(_{\text{GFP-nb}}) = 0.34$, $P(_{\text{GFP-nb-KS}}) = 0.55$, $P(_{\text{GFP-nb-KS-F-to-G}}) = 0.37$. "n" represents number of cells as in **e** examined over one experiment. The experiment was repeated independently three times with similar results.

Supplementary Figure 6

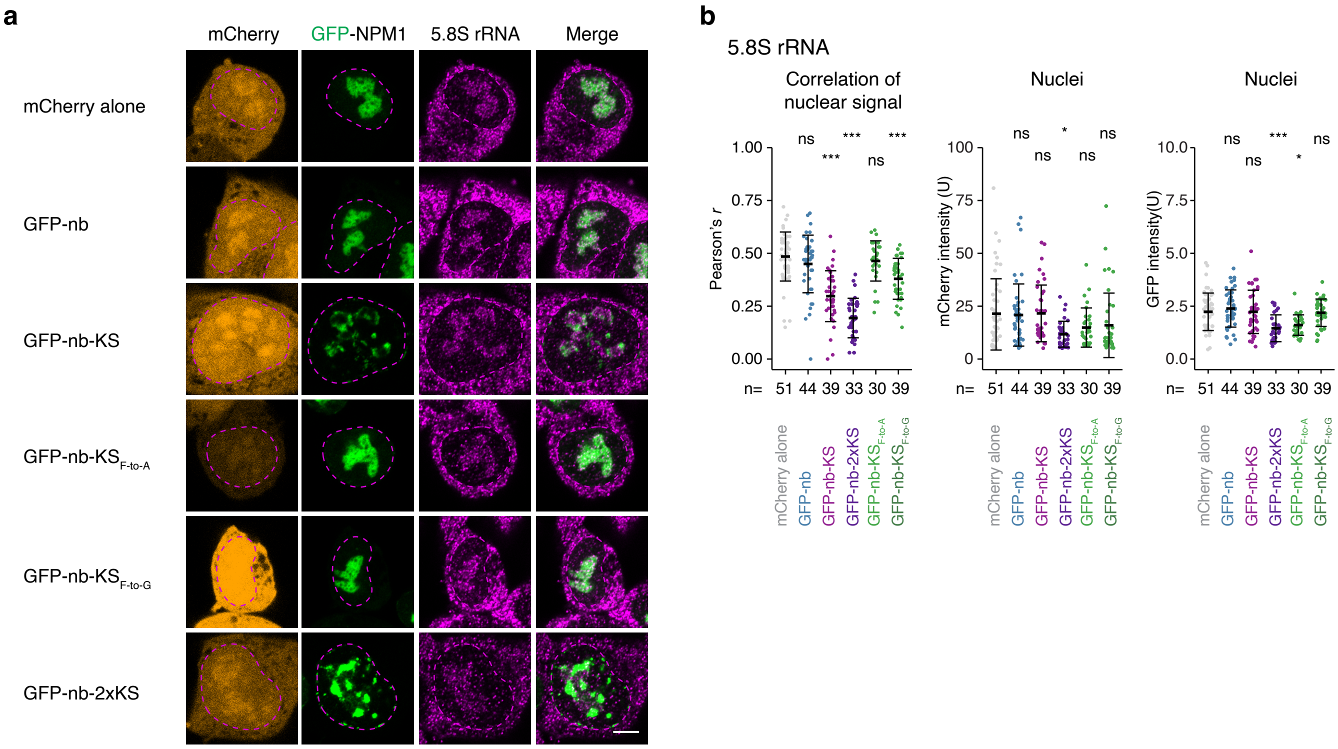

Supplementary Figure 6. Arrested dynamics of NPM1 in nucleoli impairs rRNA partitioning.

a. Fixed cell immunofluorescence for 5.8S rRNA in HCT116 cells expressing GFP-NPM1 from endogenous locus and ectopic GFP-nb constructs with cleaved mCherry reporter. Scale bar: 5μm. The experiment was repeated independently twice with similar results.

b. Correlation of GFP-NPM1 and 5.8S rRNA staining intensities measured by Pearson's correlation coefficients (left) and quantifications of mean nuclear mCherry (middle) and GFP (right) intensities. Each dot represents one nucleus. Mean is shown as a black line and whiskers represent standard deviation. "n" represents number of cells examined over one experiment. The experiment was repeated independently twice with similar results. *P*-values are from Tukey's post-hoc test versus mCherry-alone after one-way ANOVA. (Left): $P_{(\text{GFP-nb})} = 0.65$, $P_{(\text{GFP-nb-KS})} = <0.001$, $P_{(\text{GFP-nb-2xKS})} = <0.001$, $P_{(\text{GFP-nb-KS-F-to-A})} = 0.97$, $P_{(\text{GFP-nb-KS-F-to-G})} = <0.001$; (middle): $P_{(\text{GFP-nb})} = 0.99$, $P_{(\text{GFP-nb-KS})} = 0.999$, $P_{(\text{GFP-nb-2xKS})} = 0.012$, $P_{(\text{GFP-nb-KS-F-to-A})} = 0.19$, $P_{(\text{GFP-nb-KS-F-to-G})} = 0.26$; (right): $P_{(\text{GFP-nb})} = 0.94$, $P_{(\text{GFP-nb-KS})} = 1.0$, $P_{(\text{GFP-nb-2xKS})} = <0.001$, $P_{(\text{GFP-nb-KS-F-to-A})} = 0.01$, $P_{(\text{GFP-nb-KS-F-to-G})} = 0.9998$.

Supplementary Figure 7

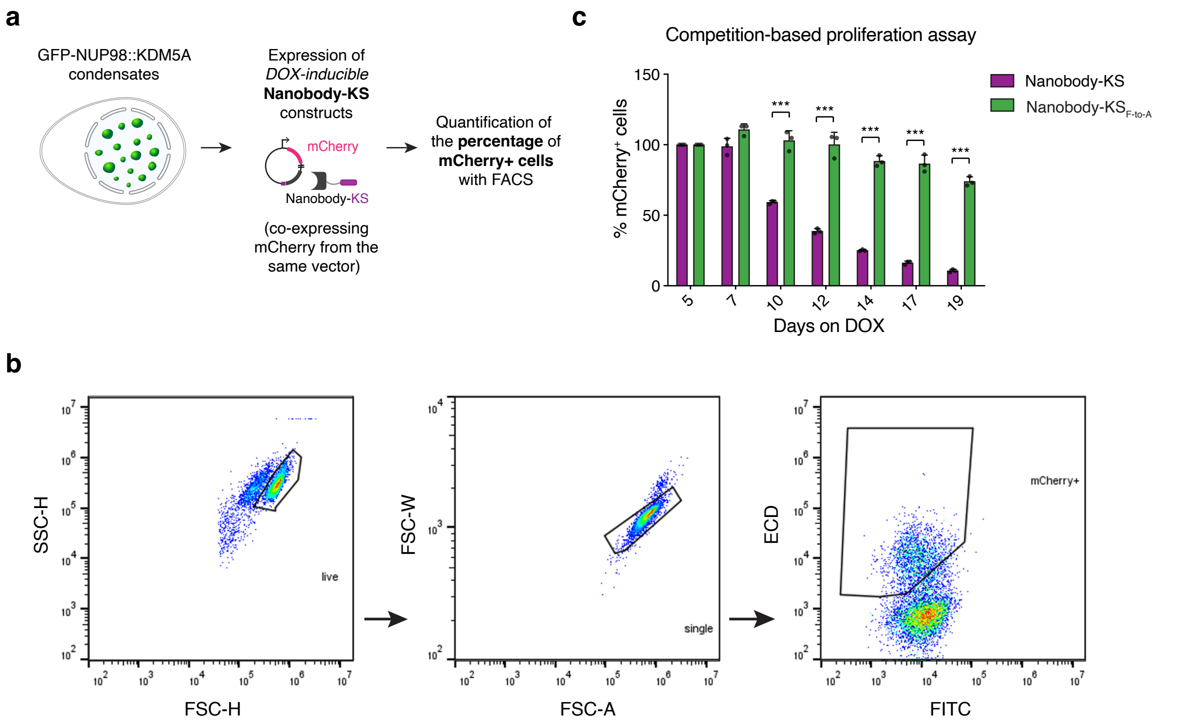

Supplementary Figure 7. Targeting the killswitch to NUP98::KDM5A condensates impairs proliferation of a GFP-NUP98::KDM5A-driven AML cell line.

a. Schematic of the experiment using αGFP-Nanobody-based setup to recruit KS to target GFP-NUP98::KDM5A.

b. Flow cytometry gating strategy for competitive cell proliferation assays in **c**.

c. Quantification of mCherry+ cells in GFP-NUP98::KDM5A AML cell line expressing N-terminally GFP-tagged NUP98::KDM5A after 5 to 19 days of doxycycline (DOX) induction. Data are presented as mean values +/- SD. P-values are from unpaired two-tailed t test. $P_{(10\ \mathrm{days})} = 0.0004$, $P_{(12\ \mathrm{days})} = 0.0003$, $P_{(14\ \mathrm{days})} = <0.0001$, $P_{(17\ \mathrm{days})} = <0.0001$, $P_{(19\ \mathrm{days})} = <0.0001$. n=3 biologically independent experiments. The statistical tests were performed on the three values of the three independent experiments.

Supplementary Figure 8

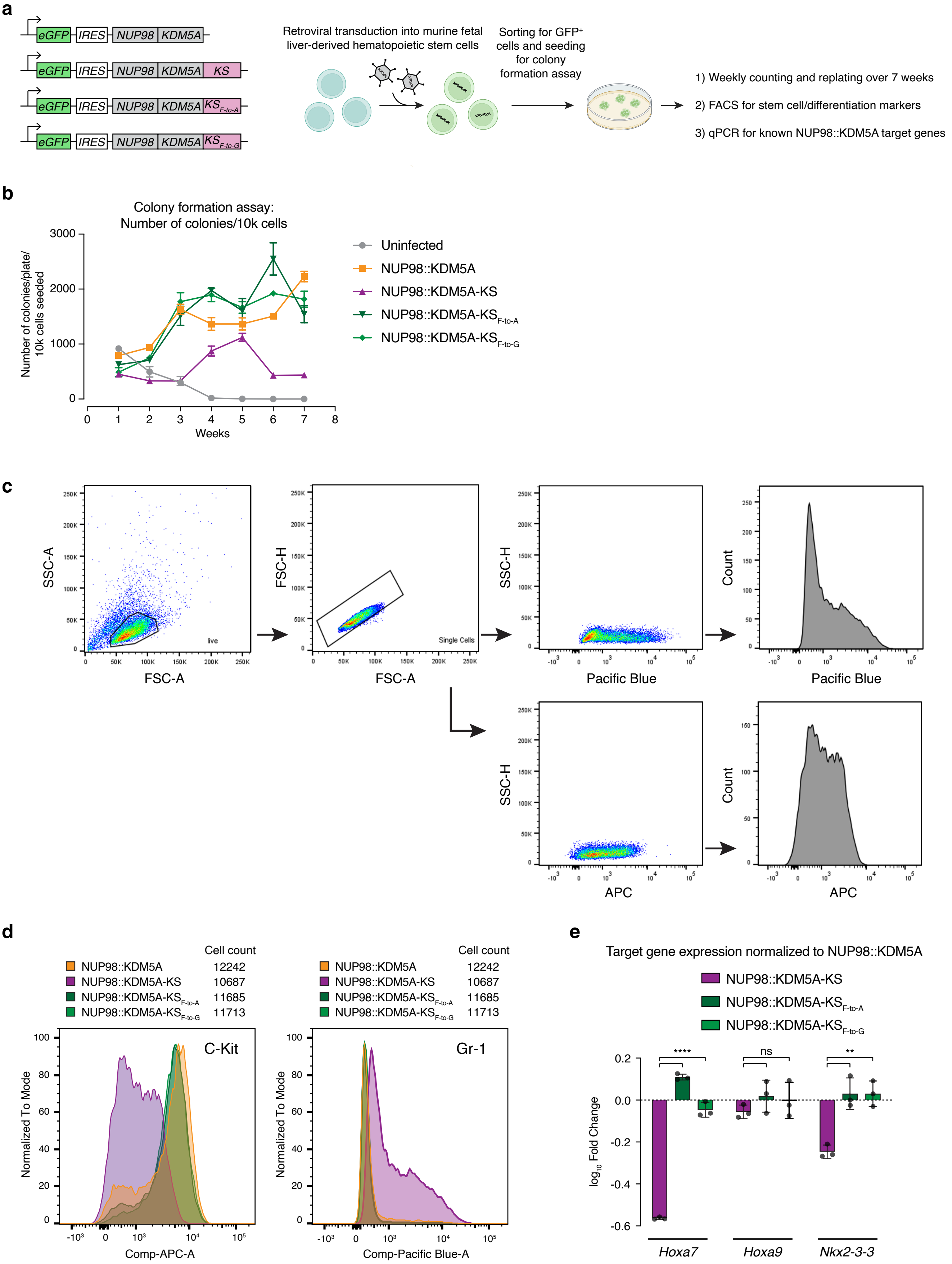

Supplementary Figure 8. Tethering the killswitch to NUP98::KDM5A impairs transformation of HPSCs to AML.

a. Schematic of the experiment to express NUP98::KDM5A-WT and -KS variants in murine fetal liver-derived hematopoietic stem cells.

b. Quantification of the number of colonies per plate formed by cells expressing NUP98::KDM5A-WT or -KS variants after 1–7 weeks. Data are presented as mean values +/- SD. n = 3 biologically independent experiments.

c. Flow cytometry gating strategy for cellular staining of anti-mouse Gr-1/Ly-6C BV421 (clone RB6-8C5) and anti-mouse CD117/c-Kit APC (clone 2B8) quantified on a FACS Canto II flow cytometer for **d**.

d. FACS quantification of C-Kit and Gr-1 expression in murine liver-derived HPSCs after the 6th round of replating of cells expressing NUP98::KDM5A and -KS variants.

e. Measurement of NUP98::KDM5A target gene expression by RT-qPCR. Data shown as $\log_{10}$ fold change. Data are presented as mean values +/- SD. n=3 technical replicates. *P*-values are from Dunnett's post-hoc testing versus NUP98::KDM5A-KS after one-way ANOVA. For *Hoxa7*, $P_{(\text{KS-FtoA})}$ = <0.0001, $P_{(\text{KS-FtoG})}$ = <0.0001; for *Hoxa9*, $P_{(\text{KS-FtoA})}$ = 0.37, $P_{(\text{KS-FtoG})}$ = 0.57; for *Nkx2-3-3*, $P_{(\text{KS-FtoA})}$ = 0.0021, $P_{(\text{KS-FtoG})}$ = 0.0021.

Supplementary Figure 9

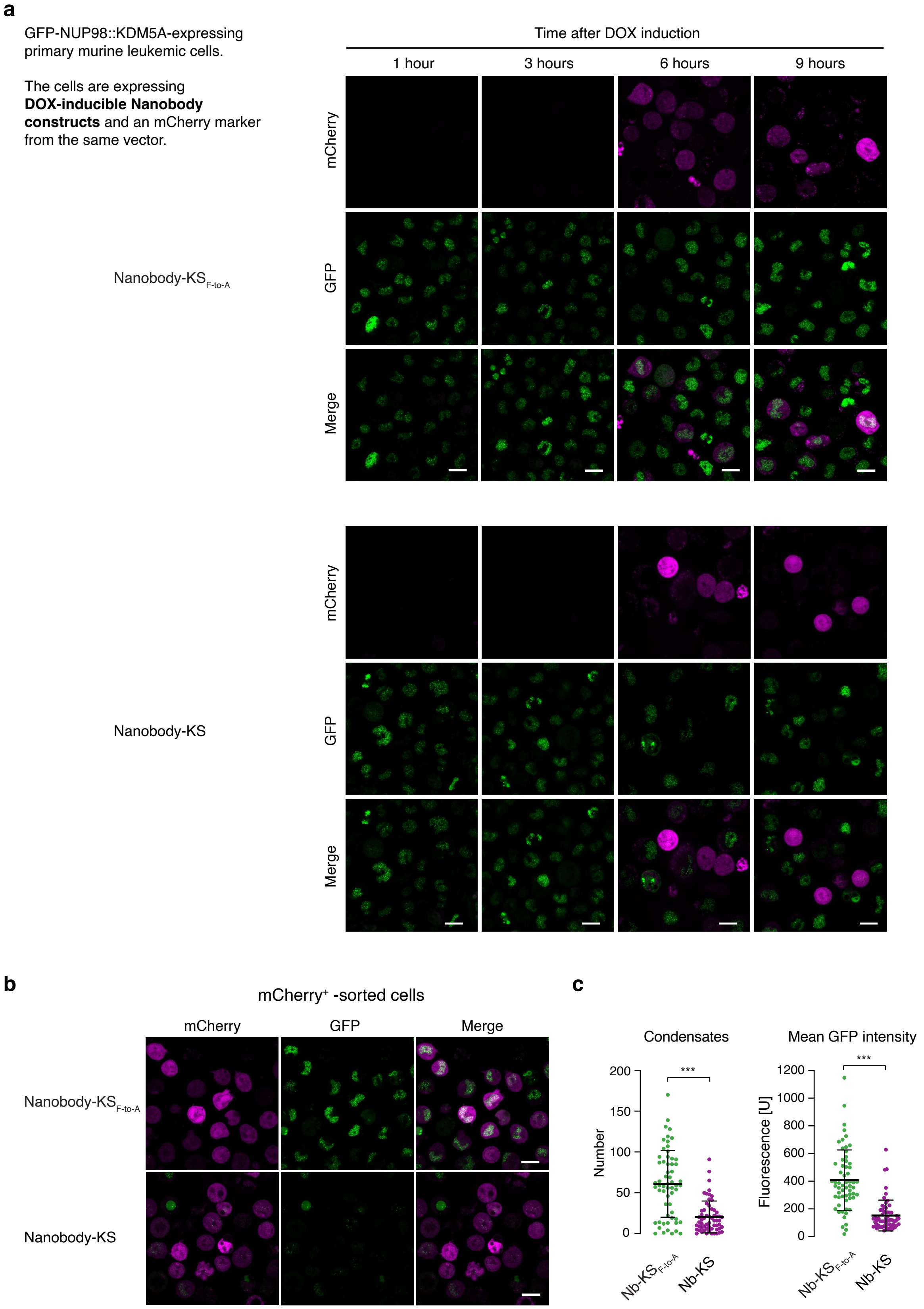

Supplementary Figure 9. GFP-NUP98::KDM5A is rapidly degraded after targeting the killswitch to NUP98::KDM5A condensates.

a. Live-cell fluorescence microscopy images of GFP-NUP98::KDM5A condensates in GFP-NUP98::KDM5A AML cells expressing Doxycycline-inducible Nanobody-$KS_{F\text{-}to\text{-}A}$ or Nanobody-KS, after the indicated hours of Dox-induction. The experiments were repeated independently three times with similar results.

b. Live-cell fluorescence microscopy images of GFP-NUP98::KDM5A condensates in sorted mCherry+ GFP-NUP98::KDM5A AML cells expressing Doxycycline-inducible Nanobody-$KS_{F\text{-}to\text{-}A}$ or Nanobody-KS, after the 9 hours of Dox-induction. Scale bar: 5 μm.

c. Quantification of number of GFP-NUP98::KDM5A condensates and mean GFP intensity in sorted mCherry+ GFP-NUP98::KDM5A AML cells expressing Doxycycline-inducible Nanobody-$KS_{F\text{-}to\text{-}A}$ or Nanobody-KS, after the 9 hours of Dox-induction. Data are presented as mean values +/- SD. Each dot represents one nucleus, n = 60 for all samples. *P*-values are from unpaired two-tailed t test. *P*-values for condensate plot and mean GFP intensity plot are <0.0001 and <0.0001, respectively.

Supplementary Figure 10

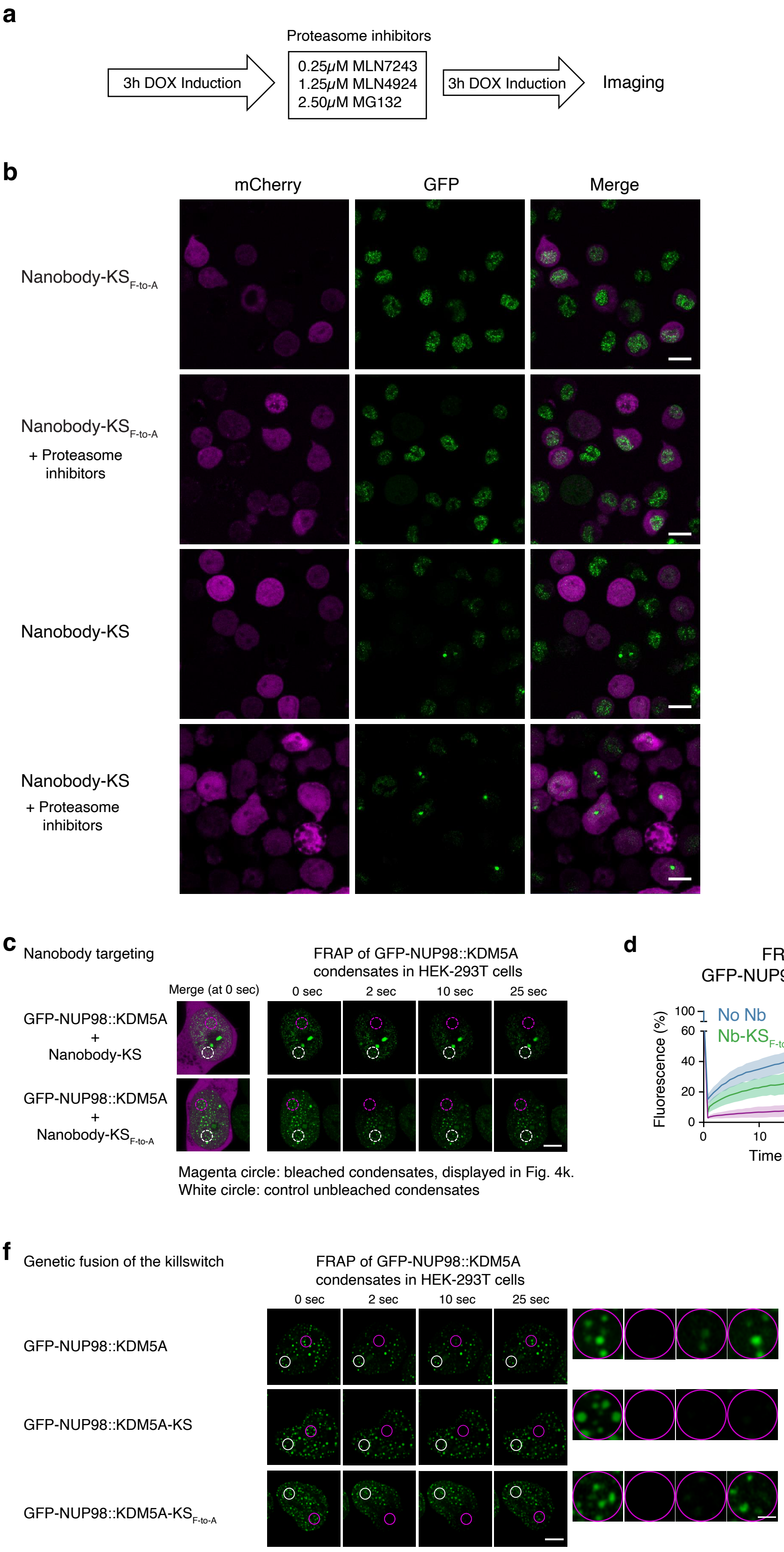

Supplementary Figure 10. Proteasome inhibitors partially rescue GFP-NUP98::KDM5A expression level.

a. Schematic of the experiment to introduce three proteasome inhibitors while inducing GFP-NUP98::KDM5A expression.

b. Live-cell fluorescence microscopy images of GFP-NUP98::KDM5A condensates in GFP-NUP98::KDM5A AML cells expressing Doxycycline-inducible Nanobody-$KS_{F\text{-}to\text{-}A}$ or Nanobody-KS, after 6h of Dox-induction. Scale bar: 5 µm. The experiments were repeated independently three times with similar results.

c. FRAP experiment in HEK-293T expressing ectopic GFP-NUP98::KDM5A and Doxycycline-inducible Nanobody-$KS_{F\text{-}to\text{-}A}$ or Nanobody-KS, after 6 hours of Dox-induction. Magenta dashed line represents bleached area and white dashed line represents photobleaching compensation area. Scale bar: 10 µm.

d. FRAP experiment in HEK-293T expressing ectopic GFP-NUP98::KDM5A and Doxycycline-inducible Nanobody-KSF-to-A or Nanobody-KS, after 6h of Dox-induction. Line displays the mean and shaded color +/- standard deviation. n = 10 for all samples.

e. Quantification of mean GFP intensity within bleached area of cells quantified in **d**. Mean is shown as a black line and whiskers represent standard deviation. *P*-values are from Tukey's multiple comparison test after one-way ANOVA. $P_{(\text{No Nb vs Nb-KS})}$ = 0.008, $P_{(\text{No Nb vs Nb-KS-F-to-A})}$ = 0.08, $P_{(\text{Nb-KS vs Nb-KS-F-to-A})}$ = 0.55.

f. Live cell fluorescence microscopy images from FRAP experiment with HEK-293T cells expressing ectopic GFP-NUP98::KDM5A with or without C-terminal fusion of KS or $KS_{F\text{-}to\text{-}A}$ variant. Magenta dashed line represents bleached area and white dashed line represents photobleaching compensation area. Scale bar: 5 µm (1 µm for zoom-in images).

g. FRAP experiment with HEK-293T cells expressing ectopic GFP-NUP98-KDM5A with or without C-terminal fusion of KS or $KS_{F\text{-}to\text{-}A}$ variant. Line displays the mean and shaded color +/- standard deviation. n = 10 for all samples.

h. Quantification of mean GFP intensity within bleached area of cells quantified in **g**. Mean is shown as a black line and whiskers represent standard deviation. *P*-values are from Tukey's multiple comparison test after one-way ANOVA. $P_{(\text{WT vs KS})}$ = 0.52, $P_{(\text{WT vs KS-F-to-A})}$ = 0.84, $P_{(\text{KS vs KS-F-to-A})}$ = 0.86. ns: not significant.

Supplementary Figure 11

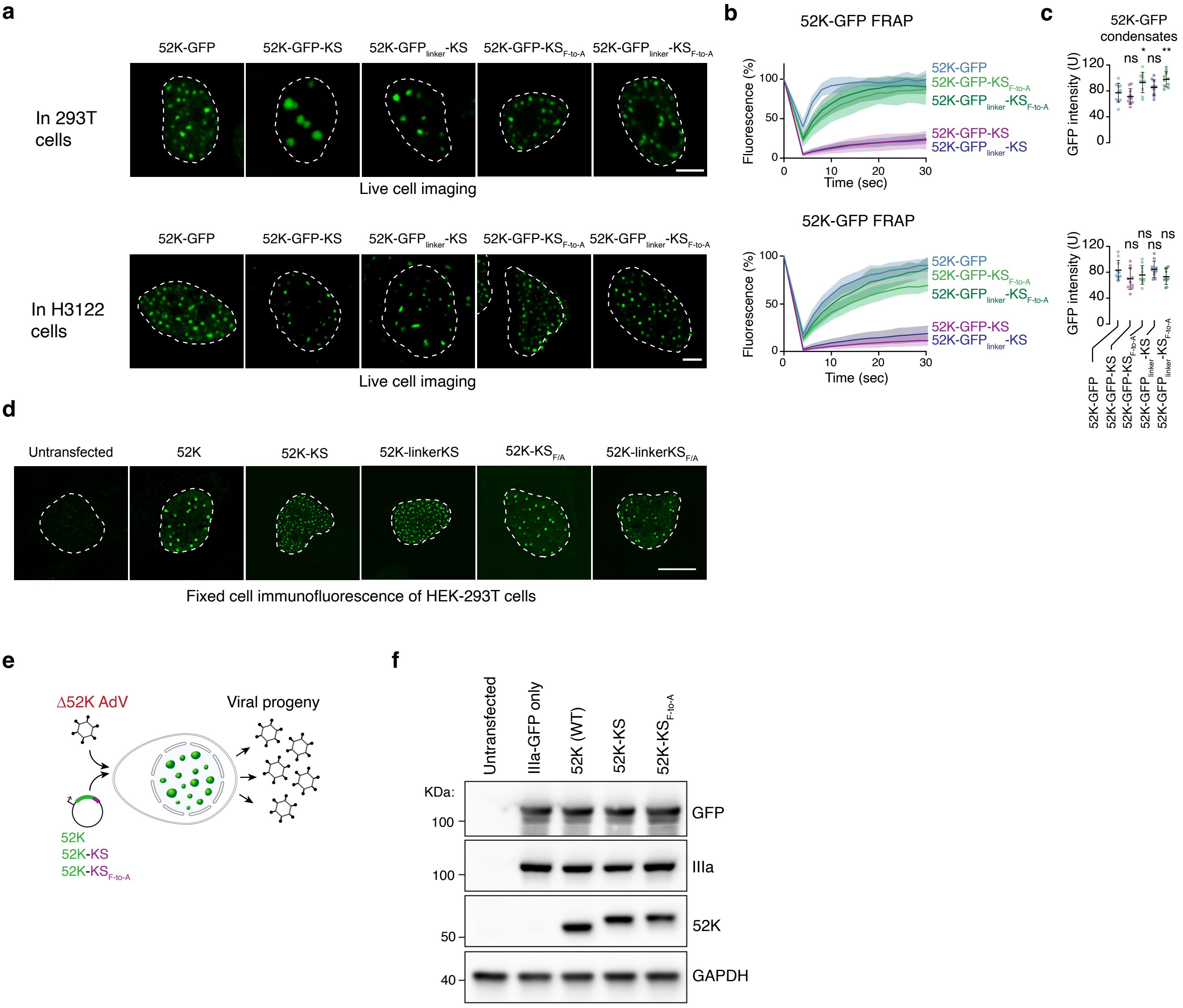

Supplementary Figure 11. The killswitch arrests dynamics of 52K condensates.

a. Live cell fluorescence microscopy images of (top) HEK-293T cells and (bottom) H3122 cells expressing ectopic 52K-GFP and 52K-GFP-KS variants. The cell nucleus is highlighted with a dashed white line contour. Scale bar: 5µm.

b. FRAP experiment for 52K-GFP from (top) HEK-293T cells and (bottom) H3122 cells expressing ectopic 52K-GFP and 52K-GFP-KS variants. Line displays the mean and shaded color +/- standard deviation. Scale bar: 5 µm.

c. Quantification of mean GFP fluorescence intensity within bleached area measured in **b**. Line displays the mean and whiskers represent standard deviation. *P*-values are from Dunnett's multiple comparisons test versus 52K-GFP after one-way ANOVA. (Top): $P_{(52K\text{-}GFP\text{-}KS)} = 0.74$, $P_{(52K\text{-}GFP\text{-}KS\text{-}F\text{-}to\text{-}A)} = 0.03$, $P_{(52K\text{-}GFP\text{-}linker\text{-}KS)} = 0.45$, $P_{(52K\text{-}GFP\text{-}linker\text{-}KS\text{-}F\text{-}to\text{-}A)} = 0.004$; (bottom): $P_{(52K\text{-}GFP\text{-}KS)} = 0.14$, $P_{(52K\text{-}GFP\text{-}KS\text{-}F\text{-}to\text{-}A)} = 0.61$, $P_{(52K\text{-}GFP\text{-}linker\text{-}KS)} = 0.998$, $P_{(52K\text{-}GFP\text{-}linker\text{-}KS\text{-}F\text{-}to\text{-}A)} = 0.35$. n = 10 cells for all samples from two biologically independent experiments.

d. Representative fixed cell immunofluorescence images from HEK293T cells expressing ectopic 52K or 52K-KS variants. The cell nucleus is highlighted with a dashed white line contour. The experiments were repeated independently twice with similar results. Scale bar: 5µm.

e. Schematic model for complementation experiment to measure viral progeny production in HEK-293T cells transduced with adenovirus lacking 52K (Δ52K) and transfected with vector encoding 52K or 52K-KS variants.

f. Western blot showing similar levels of 52K-proteins and viral structural proteins in the complementation experiment. The experiments were repeated independently twice with similar results.

Supplementary Figure 12

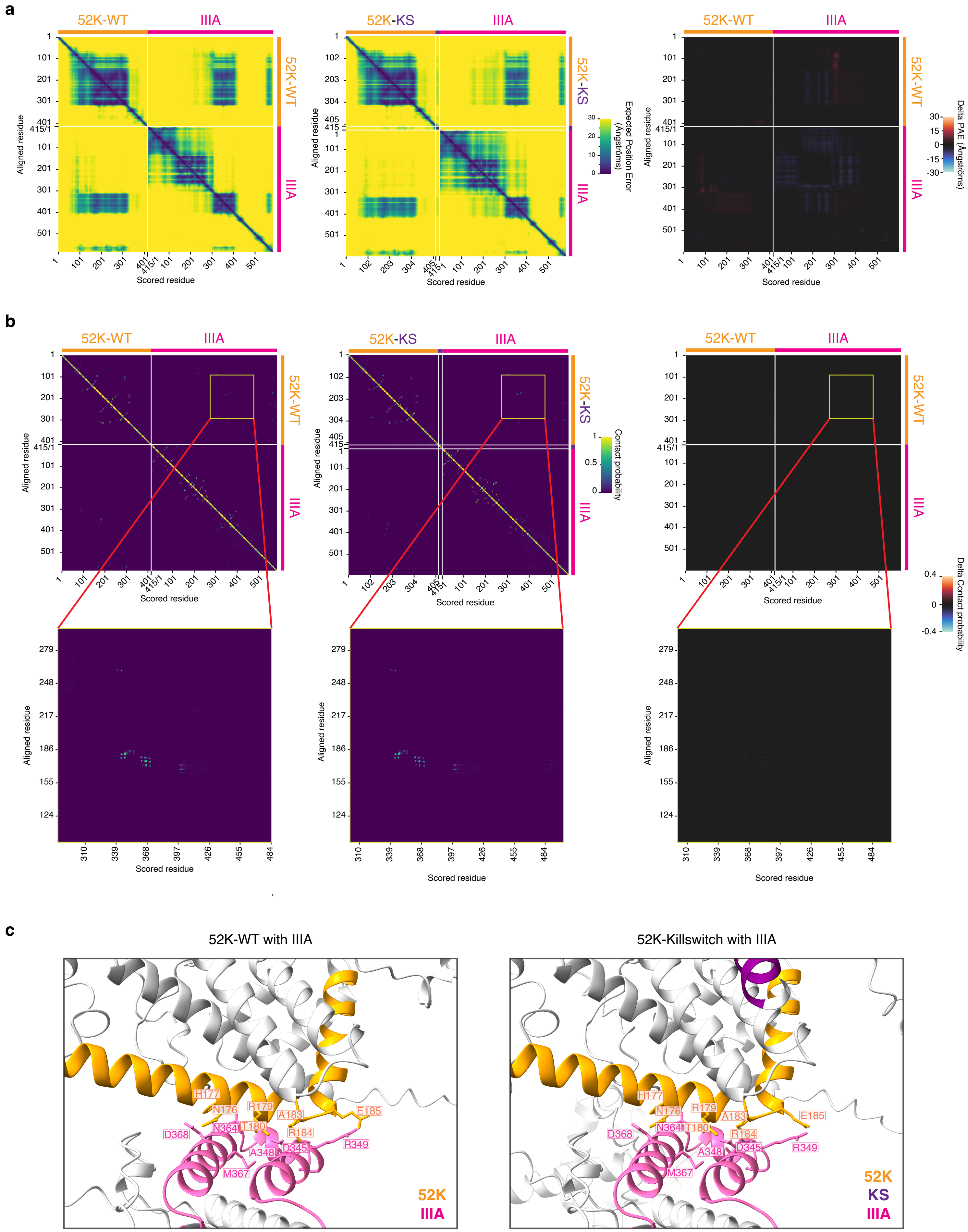

Supplementary Figure 12. Alphafold 3 models of the interaction between 52K and IIIa.

a. Predicted Alignment Error (PAE) heatmaps for multimer structure predictions by Alphafold 3 for (left) 52K-WT with IIIA, (middle) 52K-KS with IIIA, (right) Delta plot of the PAE heatmaps subtracting the 52K-WT with IIIA matrix from the 52K-KS with IIIA matrix, filtered for killswitch values. Note that there are virtually no structural change predicted in either of 52K and IIIa when the Killswitch is added to the C-terminus of 52K.

b. Contact probability prediction (CPP) heatmaps for multimer structure predictions by Alphafold 3 for (top left) 52K-WT with IIIA, (top middle) 52K-KS with IIIA, (top right) Delta plot of the CPP heatmaps subtracting the 52K-WT with IIIA matrix from the 52K-KS with IIIA matrix, filtered for killswitch values. (bottom) Zoom-in on the contact interface between two proteins. Note that in the subtracted heatmap virtually no change in the interaction between the two proteins are predicted.

c. AlphaFold 3 predicted structure interface between 52K and IIIA with amino acid positions shown in the zoom-in of panel **b**. (left) Structure interface between 52K-WT and IIIA, (right) structure interface between 52K-KS and IIIA. The contact amino acids between the two proteins with contact probability higher than 0.6 are annotated.

Supplementary Figure 13

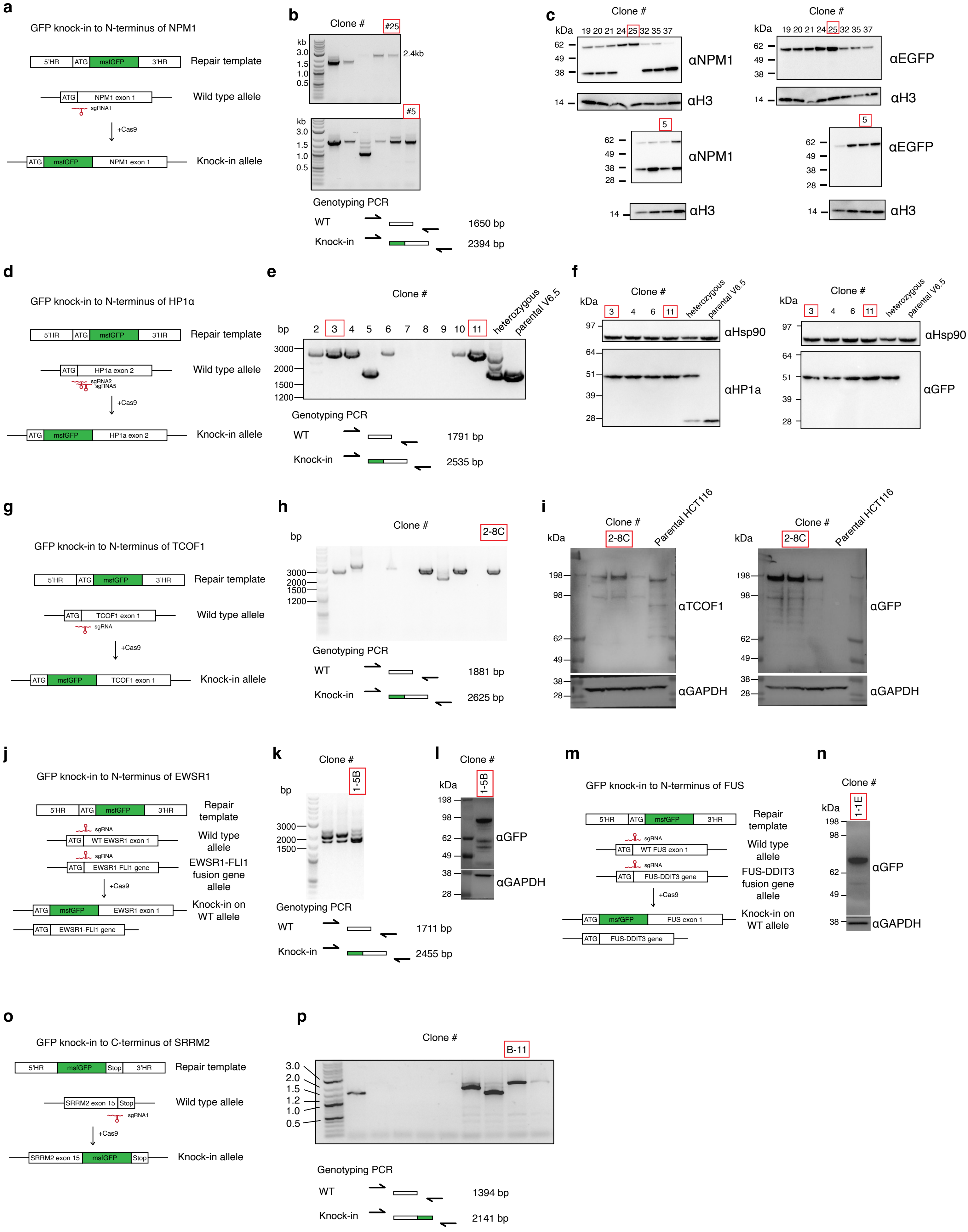

Supplementary Figure 13. Genotyping of GFP knock-in clones.

a. Scheme for GFP knock-in strategy at NPM1 locus.

b. Genotyping PCR result for HCT-116 (top, clone #25) and U2OS (bottom, clone #5) NPM1 GFP knock-in clones. Clone used in this study is highlighted with a red box. Scheme for genotyping strategy and amplicon sizes are shown below the image.

c. Western blots on HCT-116 (top) and U2OS (bottom) NPM1 GFP knock-in clones. Clones used in this study are highlighted with a red box.

d. Scheme for GFP knock-in strategy at HP1α locus.

e. Genotyping PCR results for mESC HP1α GFP knock-in clones. Clones used in this study are highlighted with a red box. Scheme for genotyping strategy and amplicon sizes are shown below the image.

f. Western blots on mES cell HP1α GFP knock-in clones. Clone used in this study is highlighted with a red box.

g. Scheme for GFP knock-in strategy at TCOF1 locus.

h. Genotyping PCR results for HCT-116 TCOF1 GFP knock-in clones. Clone used in this study is highlighted with a red box. Scheme for genotyping strategy and amplicon sizes are shown below the image.

i. Western blots on HCT-116 TCOF1 GFP knock-in clones. Clone used in this study is highlighted with a red box.

j. Scheme for GFP knock-in strategy at EWSR1 locus.

k. Genotyping PCR results for TC71 EWSR1 GFP knock-in clones. Clone used in this study is highlighted with a red box. Scheme for genotyping strategy and amplicon sizes are shown below the image.

l. Western blots on TC71 EWSR1 GFP knock-in clones. Clone used in this study is highlighted with a red box.

m. Scheme for GFP knock-in strategy at FUS locus.

n. Western blots on 1765-92 FUS GFP knock-in clones. Clone used in this study is highlighted with a red box.

o. Scheme for GFP knock-in strategy at SRRM2 locus.

p. Genotyping PCR results for HCT-116 SRRM2 GFP knock-in clones. Clone used in this study is highlighted with a red box. Scheme for genotyping strategy and amplicon sizes are shown below the image.

Supplementary Figure 14

Uncropped blot images for Supplementary Figure 11f

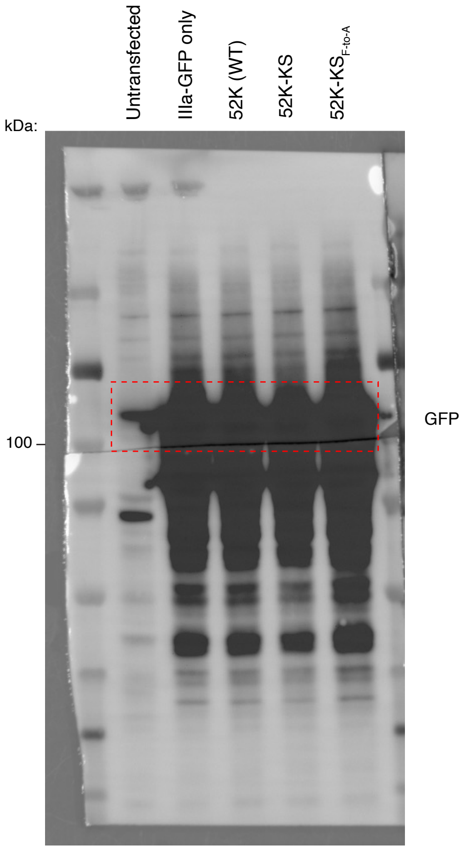

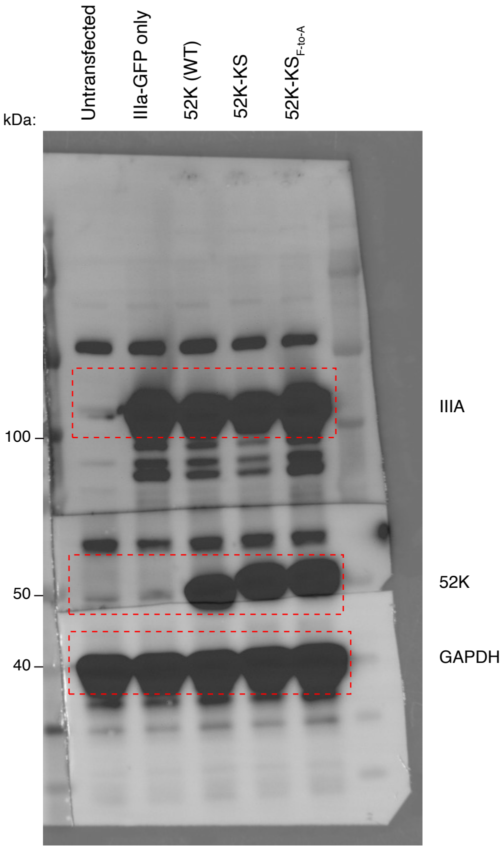

Supplementary Figure 14. Uncropped blot images for Fig. S11f.

Uncropped blot images for Supplementary Figure 11f.

Supplementary Figure 15

Uncropped blot images for Supplementary Figure 13c

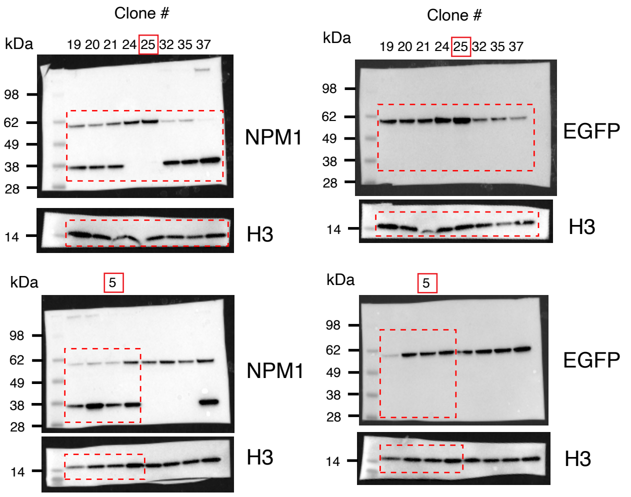

Uncropped blot images for Supplementary Figure 13f

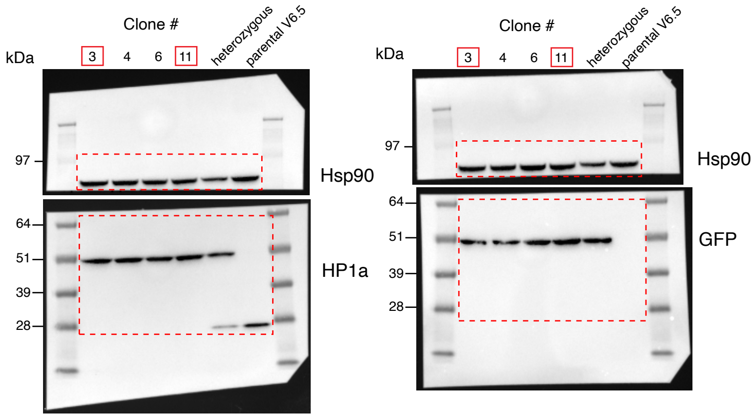

Uncropped blot images for Supplementary Figure 13i

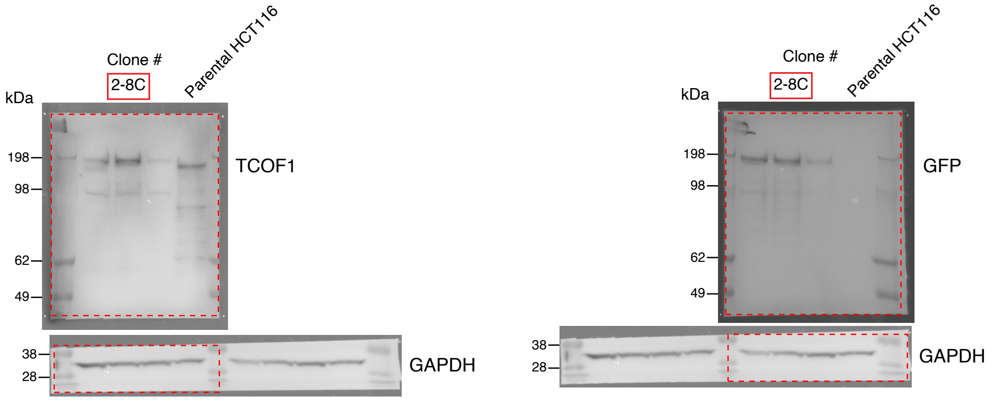

Uncropped blot images for Supplementary Figure 13l

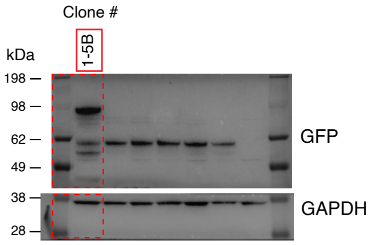

Uncropped blot images for Supplementary Figure 13n

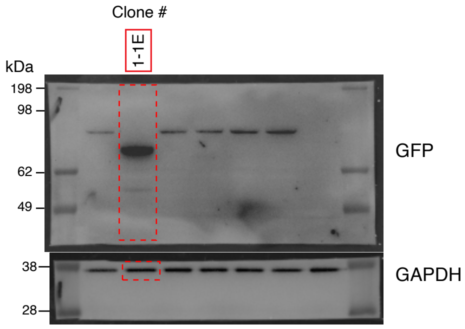

Supplementary Figure 15. Uncropped blot images for Fig. S13.

Uncropped blot images for Supplementary Figure 13.
