# Supplementary material for: Probing condensate microenvironments with a micropeptide killswitch
Source: Nature. 2025 Jun 4;643(8073):1107–16. doi: 10.1038/s41586-025-09141-5 (PMC12286862; doi:10.1038/s41586-025-09141-5)
Supplement: Supplementary file 2 — Reporting Summary [file 41586_2025_9141_MOESM2_ESM.pdf]

nature portfolio

Corresponding author(s): Henri Niskanen, Denes Hnisz

Last updated by author(s): May 2, 2025

Reporting Summary

Nature Portfolio wishes to improve the reproducibility of the work that we publish. This form provides structure for consistency and transparency in reporting. For further information on Nature Portfolio policies, see our Editorial Policies and the Editorial Policy Checklist.

Statistics

For all statistical analyses, confirm that the following items are present in the figure legend, table legend, main text, or Methods section.

n/a	Confirmed	
☐	☒	The exact sample size (*n*) for each experimental group/condition, given as a discrete number and unit of measurement
☐	☒	A statement on whether measurements were taken from distinct samples or whether the same sample was measured repeatedly
☐	☒	The statistical test(s) used AND whether they are one- or two-sided<br>*Only common tests should be described solely by name; describe more complex techniques in the Methods section.*
☒	☐	A description of all covariates tested
☐	☒	A description of any assumptions or corrections, such as tests of normality and adjustment for multiple comparisons
☒	☐	A full description of the statistical parameters including central tendency (e.g. means) or other basic estimates (e.g. regression coefficient) AND variation (e.g. standard deviation) or associated estimates of uncertainty (e.g. confidence intervals)
☐	☒	For null hypothesis testing, the test statistic (e.g. *F*, *t*, *r*) with confidence intervals, effect sizes, degrees of freedom and *P* value noted<br>*Give P values as exact values whenever suitable.*
☒	☐	For Bayesian analysis, information on the choice of priors and Markov chain Monte Carlo settings
☒	☐	For hierarchical and complex designs, identification of the appropriate level for tests and full reporting of outcomes
☐	☒	Estimates of effect sizes (e.g. Cohen's *d*, Pearson's *r*), indicating how they were calculated

Our web collection on statistics for biologists contains articles on many of the points above.

Software and code

Policy information about availability of computer code

Data collection	Data from fluorescence microscopy was acquired with Zen Black 2.3 (Zeiss).
Data analysis	Microscopy data was analyzed using ImageJ 2.14.0/1.54f or ZenBlue 3.2, 3.4, or 3.9 (Zeiss) as indicated in methods. Graph generation and statistical analysis was performed using GraphPad Prism v9 and Rstudio 2024.04.2 with multicomp and ggplot 2 packages. Protein structure predictions were performed using AlphaFold v3. Structural data was visualized using ChimeraX v1.6. Proteomics raw peak data was processed with MaxQuant v2.6.6.0 and output processed with Alphastats v0.6.9. Proteomics correlation calculations were done using SciPy packagev1.10.1 in python v3.10, volcano plots with Alphastats, and plotted with seaborn v0.13.2. RNA-Seq data was trimmed with TrimGalore v0.6.10, mapped using STAR v 2.7.11b and differential gene analysis was done using DEseq2 v1.42.1. Data visualization with ggplot2, distance matrix using dist function, R v4.4. Flow Cytometry data was collected and analysed using BD FACSDiva™ Software vs 8.0.1 or ForeCyte Software. Growth curves were recorded using ForeCyte Software (Essen Bioscience, Ann Arbor, Michigan, USA) Standard Edition 10.0 (R1) Version 10.0.8272, Build Date 8/25/2022.<br><br>All custom code used for data analyses are publicly available at Zenodo under https://doi.org/10.5281/zenodo.15322636.

For manuscripts utilizing custom algorithms or software that are central to the research but not yet described in published literature, software must be made available to editors and reviewers. We strongly encourage code deposition in a community repository (e.g. GitHub). See the Nature Portfolio guidelines for submitting code & software for further information.

Data

Policy information about availability of data

All manuscripts must include a data availability statement. This statement should provide the following information, where applicable:

- Accession codes, unique identifiers, or web links for publicly available datasets
- A description of any restrictions on data availability
- For clinical datasets or third party data, please ensure that the statement adheres to our policy

Sequencing data were deposited at the Gene Expression Omnibus, under the accession ID: GSE284494. Mass spectrometry data were deposited to the ProteomeXchange Consortium (http://proteomecentral.proteomexchange.org) via the PRIDE partner repository, with the dataset identifier PXD058854. The NGS experiments of human samples used the human genome hg38 and annotation from GENCODE GRCh38.p13. Plasmids were deposited at Addgene (237619-237693 and 238231-238298). All raw and processed data were deposited at Zenodo, and are publicly available under https://doi.org/10.5281/zenodo.15322636. The source data behind all graphs in figures are provided with this paper as a Source Data workbook.

Research involving human participants, their data, or biological material

Policy information about studies with human participants or human data. See also policy information about sex, gender (identity/presentation), and sexual orientation and race, ethnicity and racism.

Reporting on sex and gender	No data on human participants is included.
Reporting on race, ethnicity, or other socially relevant groupings	No data on human participants is included.
Population characteristics	No data on human participants is included.
Recruitment	No data on human participants is included.
Ethics oversight	No data on human participants is included.

Note that full information on the approval of the study protocol must also be provided in the manuscript.

Field-specific reporting

Please select the one below that is the best fit for your research. If you are not sure, read the appropriate sections before making your selection.

☒ Life sciences ☐ Behavioural & social sciences ☐ Ecological, evolutionary & environmental sciences

For a reference copy of the document with all sections, see nature.com/documents/nr-reporting-summary-flat.pdf

Life sciences study design

All studies must disclose on these points even when the disclosure is negative.

Sample size	No statistical methods were used to predetermine sample sizes. Sample sizes for RNA, protein expression and imaging experiments are consistent with current standards and are indicated in the figures, legends or methods section. Examples of imaging & FRAP: PMID: 37683610; RNA-seq: PMID: 39373104, PMID: 39448850, proteomics: PMID: 38355802.
Data exclusions	Thresholds of mCherry expression were used to exclude non-transfected cells as described in the methods. We used cleavable mCherry as a reporter to focus analysis on cells that had been transfected, and omitted untransfected cells based on mCherry expression level. Thresholds of mCherry expression were not pre-determined, but determined empirically based on each experiment and are described in the methods section. Although threshold vary between different experimental and image acquisition conditions, thresholds were kept similar across samples that were compared. In addition, for BRD4::NUT hexanediol experiment, nuclei with saturated pixels on GFP channel were excluded to have reliable measurements of GFP signal variability.
Replication	All experiments were repeated at least two times with similar results unless stated otherwise in the figure legends or “Statistics and Reproducibility” in the methods section.
Randomization	Not relevant for this study. Samples were allocated into wt, KS and KS variant groups without randomization, but the order of samples on plates or in which they are imaged would not affect the results.
Blinding	Blinding was not relevant for these experiments and in some cases even impossible since solidifying effect of the KS would become apparent during imaging. However, data was collected and analyzed in similar manner regardless of sample groups, allowing unbiased analysis of data.

Reporting for specific materials, systems and methods

We require information from authors about some types of materials, experimental systems and methods used in many studies. Here, indicate whether each material, system or method listed is relevant to your study. If you are not sure if a list item applies to your research, read the appropriate section before selecting a response.

Materials & experimental systems

n/a	Involved in the study
☐	☒ Antibodies
☐	☒ Eukaryotic cell lines
☒	☐ Palaeontology and archaeology
☐	☒ Animals and other organisms
☒	☐ Clinical data
☒	☐ Dual use research of concern
☒	☐ Plants

Methods

n/a	Involved in the study
☒	☐ ChIP-seq
☐	☒ Flow cytometry
☒	☐ MRI-based neuroimaging

Antibodies

Antibodies used

Alexa Fluor 647 donkey anti-mouse, Jackson Immuno Research, 715-605-150, 1:1000.
Alexa Fluor 647 anti-rabbit, Jackson Immuno Research, and 711-605-152, 1:1000.
5.8S rRNA (Novus, NB100-662SS, 1:500)
RNAPII (Abcam, ab26721, 1:500)
H3K27Ac (Abcam, ab4729, 1:1000)
Alexa Fluor goat anti-rabbit 488 antibody (Life Technologies, Cat#: A-11008), 1:1000.
goat anti-mouse 488 antibody (Life Technologies, Cat#: A-11001), 1:1000.
Antibody to 52K (gift from P. Hearing, Stony Brook University, NY; PMID: 15709002), species: rabbit, polyclonal, 1:500.
IIIA (gift from P. Hearing), species: rabbit, polyclonal, WB 1:10,000
DBP (gift from A. Levine; PMID: 6310869), species: mouse, clone: B6-8, 1:400.
HA-Tag (C29F4) Rabbit mAb #3724, Cell Signaling, 1:1000.
Hexon, Penton, Fiber (Abcam Cat#: ab6982), species: rabbit, polyclonal, WB 1:10,000.
GAPDH (GeneTex, Cat#: GTX100118, Lot: 43712), species: rabbit, polyclonal, WB 1:5,000
HRP-conjugated goat anti-rabbit (Jackson Laboratories, Cat#: 111-035-045), 1:10,000.
TCOF1 (Santa Cruz, sc-374536, 1:750),
GFP (Invitrogen, A11122, 1:2000),
NPM1 (Invitrogen, 32-5200, 1:2000),
HP1α (CST, #2616, 1:1000),
Histone H3 (Abcam, ab1719, 1:10000),
GAPDH (CST, #14C10, 1:4000),
HSP90 (BD, 610419, 1:2000),
anti-mouse Gr-1/Ly-6C BV421 (clone RB6-8C5, Biolegend), 1:200,
anti-mouse CD117/c-Kit APC (clone 2B8, Biolegend), 1:200.
NEPRO (Santa cruz, sc-376579) 1:100

Validation

Antibodies were not validated in-house, but all antibodies have been cited in numerous publications.

5.8S rRNA (Novus, NB100-662SS)
https://www.novusbio.com/PDFs/NB100-662.pdf

RNAPII (Abcam, ab26721)
https://www.abcam.com/en-us/products/primary-antibodies/rna-polymerase-ii-ctd-repeat-ysptsps-antibody-chip-grade-ab26721?srsltid=AfmBOooZuSRt47SrB9UwBQaor2WJ_evhZYeRoKK76hbWpNqppNHsoAlb

H3K27Ac (Abcam, ab4729)
https://www.abcam.com/en-us/products/primary-antibodies/histone-h3-acetyl-k27-antibody-chip-grade-ab4729?srsltid=AfmBOorW3BjfDiqA1eFCeG6ejgg5O1n-ZQZYPUXfT4LtLa_b0djgpVi8

HA-Tag (C29F4) (#3724, Cell Signaling)
https://www.cellsignal.com/products/3724/datasheet?images=1&protocol=0&size=A4

GAPDH (GeneTex, Cat#: GTX100118)
https://www.genetex.com/PDF/Download?catno=GTX100118&srsltid=AfmBOopuq1lsZv4hVsKqaGuu4U43wwnYWWmOX0Dt4_ktlK83gTnFnBwF

TCOF1 (Santa Cruz, sc-374536)

https://datasheets.scbt.com/sc-374536.pdf

GFP (Invitrogen, A11122)
https://www.thermofisher.com/order/genome-database/dataSheetPdf?producttype=antibody&productsubtype=antibody_primary&productId=A-11122&version=Local

NPM1 (Invitrogen, 32-5200)
https://www.thermofisher.com/order/genome-database/dataSheetPdf?producttype=antibody&productsubtype=antibody_primary&productId=32-5200&version=Local

HP1α (CST, #2616)
https://www.cellsignal.com/products/2616/datasheet?images=1&protocol=0&size=A4

Histone H3 (Abcam, ab1719)
https://www.abcam.com/en-us/products/primary-antibodies/histone-h3-antibody-nuclear-marker-and-chip-grade-ab1791?srsltid=AfmBOop1I1ZVZPzH92dKeqZrPNGqTvF7LHjTFbUSfpDHbD-0iyCHFVgC

GAPDH (CST, #14C10)
https://www.cellsignal.com/products/2118/datasheet?images=1&protocol=0&size=A4

HSP90 (BD, 610419)
https://www.bdbiosciences.com/content/dam/bdb/products/global/reagents/microscopy-imaging-reagents/immunofluorescence-reagents/610xxx/6104xx/610419_base/pdf/610419.pdf

anti-mouse Gr-1/Ly-6C BV421 (clone RB6-8C5, Biolegend)
https://d1spbj2x7qk4bg.cloudfront.net/Default.aspx?ID=13406&pdf=true&displayInline=true&ProductID=460&leftRightMargin=15&topBottomMargin=15&filename=PE%20anti-mouse%20Ly-6GLy-6C%20(Gr-1)%20Antibody.pdf&v=20250407123848

anti-mouse CD117/c-Kit APC (clone 2B8, Biolegend)
https://d1spbj2x7qk4bg.cloudfront.net/Default.aspx?ID=9851&pdf=true&displayInline=true&ProductID=77&leftRightMargin=15&topBottomMargin=15&filename=Purified%20anti-mouse%20CD117%20(c-Kit)%20Antibody.pdf&v=20250407123848

NEPRO (Santa cruz, sc-376579)
https://datasheets.scbt.com/sc-376579.pdf

Adenovirus late protein antibody (gift from J. Wilson): Recognizes Hexon (band of approx. 110 kDa), Penton (band of approx. 65 kDa), and Fiber (band of approx. 61 kDa) in adenovirus infected whole cell lysates but not uninfected lysates (Kozarsky et al. 1996. DOI: 10.1038/ng0596-54; Herrmann et al. 2020. DOI: 10.1038/s41564-020-0750-9; Charman et al. 2023. DOI: 10.1038/s41586-023-05887-y).

52K (gift from P. Hearing): Recognizes 52K (band of approx. 52kDa) on western blots of adenovirus infected whole cell lysates, but not uninfected whole cell lysates or lystaes from cells infected with a Δ52K mutant adenovirus. Immunofluorescence staining shows signal in adenovirus infected cells but not uninfected cells. (Ostapchuk et al. 2005. DOI: 10.1128/JVI.79.5.2831-2838.2005; Charman et al. 2023. DOI: 10.1038/s41586-023-05887-y).

IIIa (gift from P. Hearing): Recognizes IIIa (band of approx. 65 kDa) on western blots of adenovirus infected whole cell lysates or adenovirus particles, but not uninfected whole cell lysates (Ma & Hearing. 2011. DOI: 10.1128/JVI.00467-11; Charman et al. 2023. DOI: 10.1038/s41586-023-05887-y).

DBP (gift from A. Levine): Recognizes DBP (band of approx. 72 kDa) on western blots of adenovirus infected whole cell lysates, but not uninfected whole cell lysates. Immunofluorescence staining shows signal in adenovirus infected cells but not uninfected cells. Immunofluorescence staining shows localization to viral replication compartments in H5ts107-infected cells grown at the permissive temperature of 32°C but not in H5ts107-infected cells grown at the non-permissive temperature of 39.5° C (Reich et al. 1983. DOI: 10.1016/0042-6822(83)90274-x; Charman et al. 2023. DOI: 10.1038/s41586-023-05887-y).

Eukaryotic cell lines

Policy information about cell lines and Sex and Gender in Research

Cell line source(s)

U2-OS (ATCC #HTB-96),
HEK293T (ATCC #CRL-3216),
Lenti-X 293T (Takara Bio, Cat. No. 632180),
HCT-116 (ATCC #CCL-247),
HCT-116 GFP-NPM1 (this study),
U2OS GFP-NPM1 (this study),
HCT-116 GFP-TCOF1 (this study),
HCT-116 SRRM2-GFP (this study),
HAP1 SRRM2-GFP tr0 (Source: Tugce Aktas lab, PMID: 33095160),
A673 (Source: Dr. Heinrich Kovar. Originally from CLS #300454),
A673 Dox-inducible EGFP-NPM1 (this study),
MCF7 (ATCC # HTB-22),
C2C12 (ATCC # CRL-1772),

V6.5 mESC (Source: Alexander Meissner lab),
V6.5 mESC GFP-HP1a (this study),
Murine fetal liver cells (Source: Florian Grebien lab),
H3122 (CLS, #300484),
TC71 (DMSZ, #ACC516),
TC71 GFP-EWSR1 (this study),
1765-92 (source: Dr. Pierre Åman),
1765-92 GFP-FUS (this study).
For experiments involving expression of 52K and KS variants: HEK-293 (ATCC #CRL-1573), HEK-293T (ATCC #CRL-3216).

Authentication

Cell line identity were verified using morphological characteristics, but lines have not been authenticated.

Mycoplasma contamination

All cell lines tested negative for mycoplasma using LookOut Mycoplasma PCR Detection Kit (Sigma-Aldrich, MP0035) or PCR Mycoplasma Test Kit II (Applichem, A8994). Mycoplasma testing was carried out on 0.2–1 mL of cell culture media taken from tissue culture dishes containing confluent monolayers of cells on a routine basis at least twice a year.

Commonly misidentified lines
(See ICLAC register)

No commonly misidentified cell lines were used in the study.

Animals and other research organisms

Policy information about studies involving animals; ARRIVE guidelines recommended for reporting animal research, and Sex and Gender in Research

Laboratory animals

For establishment of murine AML cell lines, fetal liver cells were transduced with oncogene-expressing plasmids and transplanted into recipient mice. For this, male and female C57BL/6J.SJL mice at the age of 10-12 weeks were used. Mice were kept in specific opportunistic pathogen free quality (SOPF) under stringent controlled standard conditions, in individually ventilated cages, fed with Sniff Haltungsfutter CHOW standard 10mm pellets (Catalog-No. V1534-000), ad libitum.
For the Zebrafish experiments: the study used embryos of Wild Type (ABTL) zebrafish. No fish older than 5 days were used, which means that according to the regulations on animal experimentation, we did not perform animal experiments. Adult fish were maintained according to local husbandry regulation, which equally does not constitute animal experimentation. To obtain embryos, male and female fish were placed in a water tank with a separating net in the afternoon; the fish were placed together the following morning and embryos could be collected after few minutes of spontaneous mating.

Wild animals

No wild animals were used in the study.

Reporting on sex

Male and female recepient mice were used in this study for transplantation and establishment of murine AML models. This study does not include any experiments in which animals were subjected to different treatment cohorts, for which sex-based analysis would be relevant.

Field-collected samples

No field collected samples were used in the study.

Ethics oversight

All animal studies were performed according to ethical animal license protocols and were approved by the responsible authorities of the Austrian government (BMBWF-68.205/0199-V/3b/2018).

Note that full information on the approval of the study protocol must also be provided in the manuscript.

Plants

Seed stocks

Not relevant for this study.

Novel plant genotypes

Not relevant for this study.

Authentication

Not relevant for this study.

Flow Cytometry

Plots

Confirm that:

☒ The axis labels state the marker and fluorochrome used (e.g. CD4-FITC).

☒ The axis scales are clearly visible. Include numbers along axes only for bottom left plot of group (a 'group' is an analysis of identical markers).

☐ All plots are contour plots with outliers or pseudocolor plots.

☒ A numerical value for number of cells or percentage (with statistics) is provided.

Methodology

Sample preparation	For NUP98::KDM5A experiments: cells were washed with PBS and resuspended in PBS with 0.5% FCS, followed by staining for 30 min with dilutions (1:200) of the following antibodies (all from Biolegend, San Diego, CA, USA): anti-mouse Gr-1/Ly-6C BV421 (clone RB6-8C5) and anti-mouse CD117/c-Kit APC (clone 2B8).<br>For NuFANCI experiments: described comprehensively in NuFANCI method section.
Instrument	For NUP98::KDM5A: BD FACSCanto II; For NuFANCI: BD FACSAria™ Fusion
Software	FlowJo (FlowJo LLC, Ashland, OR, USA).
Cell population abundance	The final sorted populations of "Nucleolus" were 13.7 - 27.4% of the total events. Representative samples of n=4 independent experiments of each condition are shown in Supplementary Figure 4e.
Gating strategy	For NuFANCI experiments, sorting of "Nucleolus" three gates were used: 1) DAPI (uv-450/50-A) vs GFP (b-530/30-A) was used to identify the population containing "Nucleolus" (GFP+, DAPI-intermediate), determined by sorting different fractions outlined in Supplementary Figure 1c and subsequent imaging (Supplementary Figure 1d); 2) FSC-A vs SSC-A gate was used to exclude large events; 3) GFP (b-530/30) vs mCherry (yg-610/20) was used to sort for either mCherry– (for the samples untransfected and Actinomycin D) or mCherry+ (for the samples Nb, KS, KSFtoG and 2xKS). Gates were determined by comparing to mCherry– samples (untransfected). For NUP98::KDM5A experiments, Live cells were discriminated based on forward scatter height (FSC-H) and side scatter height (SSC-H). Single cells were gated based on forward scatter height (FSC-H) and forward scatter area (FSC-A). mCherry-positive cells were identified by their signal intensity in the ECD channel. Cellular staining for anti-mouse Gr-1/Ly-6C was assessed based on the signal intensity in the Pacific Blue channel (BV421), while anti-mouse CD117/c-Kit staining was evaluated based on signal intensity in the APC channel.

☒ Tick this box to confirm that a figure exemplifying the gating strategy is provided in the Supplementary Information.
