# Supplementary figures and images for: Probing condensate microenvironments with a micropeptide killswitch
Source: Nature. 2025 Jun 4;643(8073):1107–16. doi: 10.1038/s41586-025-09141-5 (PMC12286862; doi:10.1038/s41586-025-09141-5)

Uncropped blot images for Figure 5f

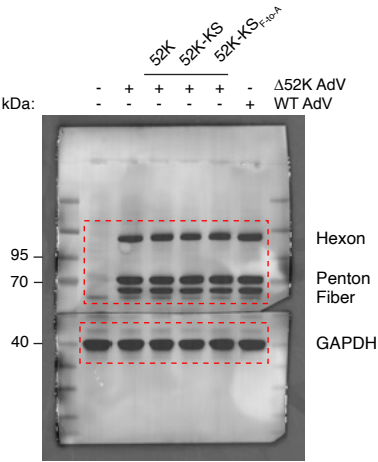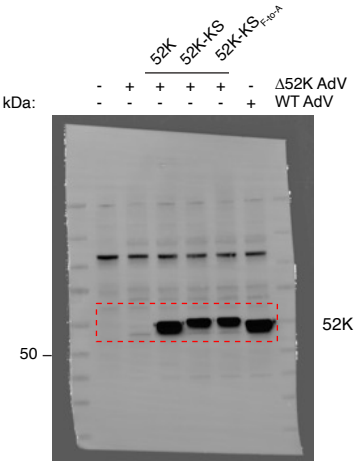

Supplement: Supplementary file 3 — Uncropped blots used in Fig 5. [file 41586_2025_9141_MOESM3_ESM.pdf]

Uncropped Coomassie gel image for Extended Data Figure 7a

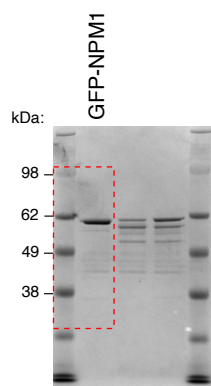

Supplement: Supplementary file 4 — Uncropped blots used in Extended Data Fig 7a. [file 41586_2025_9141_MOESM4_ESM.pdf]
